# Bear bile: dilemma of traditional medicinal use and animal protection

**DOI:** 10.1186/1746-4269-5-2

**Published:** 2009-01-12

**Authors:** Yibin Feng, Kayu Siu, Ning Wang, Kwan-Ming Ng, Sai-Wah Tsao, Tadashi Nagamatsu, Yao Tong

**Affiliations:** 1 School of Chinese Medicine, The University of Hong Kong, 10 Sassoon Road, Pokfulam, Hong Kong, PR China; 2 Department of Chemistry and Open Laboratory of Chemical Biology of the Institute of Molecular Technology for Drug Discovery and Synthesis, Faculty of Science, The University of Hong Kong, Pokfulam Road, Hong Kong, PR China; 3 Department of Anatomy, Li Ka Shing Faculty of Medicine, The University of Hong Kong, 21 Sassoon Road, Pokfulam, Hong Kong, PR China; 4 Department of Pharmacobiology and Therapeutics, Faculty of Pharmacy, Meijo University 150 Yagotoyama, Tenpakuku, Nagoya 468-8503, Japan

## Abstract

Bear bile has been used in Traditional Chinese Medicine (TCM) for thousands of years. Modern investigations showed that it has a wide range of pharmacological actions with little toxicological side effect and the pure compounds have been used for curing hepatic and biliary disorders for decades. However, extensive consumption of bear bile made bears endangered species. In the 1980's, bear farming was established in China to extract bear bile from living bears with "Free-dripping Fistula Technique". Bear farming is extremely inhumane and many bears died of illness such as chronic infections and liver cancer. Efforts are now given by non-governmental organizations, mass media and Chinese government to end bear farming ultimately. At the same time, systematic research has to be done to find an alternative for bear bile. In this review, we focused on the literature, laboratory and clinical results related to bear bile and its substitutes or alternative in English and Chinese databases. We examined the substitutes or alternative of bear bile from three aspects: pure compounds derived from bear bile, biles from other animals and herbs from TCM. We then discussed the strategy for stopping the trading of bear bile and issues of bear bile related to potential alternative candidates, existing problems in alternative research and work to be done in the future.

## Background

Bears live in all the continents except Africa, Antarctica, and Australia, and they are classified into eight species including giant panda (*Ailuropoda melanoleuca*). The largest among all is polar bear (*Thalarctos maritimus*), followed by brown bear (*Ursus arctos*), American black bear (*Ursus americanus*), Asiatic black bear (*Selenarctos thibetanus*), spectacled bear (*Tremarctos ornatus*), sloth bear (*Melursus ursinus*), and sun bear (*Helarctos malayanus*). All of these species are endangered – five are listed in Convention on International Trade in Endangered Species of Wild Fauna and Flora (CITES) Appendix I, whilst the remaining three are listed in Appendix II [1].

China was the first country utilizing bear bile and its gall bladder in traditional medicinal products and this usage was adopted by Korea and Japan [2,3] centuries ago. Today the use of Traditional Chinese Medicine (TCM) was widespread not only in Asia but also throughout Asian communities in other areas of the world, including Europe and America. Many of these consumers bought bear bile products, either because they believed it was a traditional medicine, or because the products were marketed well by local TCM pharmacies.

Bear bile and bile extraction in TCM were under the category of animal drugs. Its medicinal functions were well documented in ancient Chinese medicinal and modern Chinese medicine publications [4,5]. Some recent textbooks and books of TCM still recommend formulae containing various animal tissues such as tiger bones, antelope, buffalo or rhino horns, deer antlers, testicles and penis of the dog, bear or snake bile. Some of them have drawn attention from international [6,7]. Usually, animal tissues were combined with medical herbs to form Chinese Medicine preparations and in most of the cases, the medical use of these preparations was justified in terms of the rules of TCM. However, only a few researches have been done to prove the claimed clinical efficacy of TCM animal products. Still, J. (2003) [8] has published a review paper in discussing some related ecological, ethico-legal and health concerns about hunting, breeding and trading with endangered species, risks of transmission of zoonoses, quality of the products, and alternatives to the preparations from endangered species.

### Bear bile used in traditional chinese medicine

#### Traditional properties and actions

Bear bile (Xiongdan in Chinese) is the dried gallbladder with bile of *selenarctos thibetanus *curvier or *Ursus arctos *L. The dried gallbladder with bile from other species of Ursidae can be also used as Bear bile. Bear bile was first recorded in <Tang Ban Cao> (Newly Revised Materia Medica, Tang Dynasty, 659 A.D.), the first official book compiled and issued by the government and considered to be the first pharmacopoeia in China. From the point of view of TCM, bear bile was considered as a cold medicine, bitter in flavor, cool in nature and attributive to liver, gallbladder and heart, so it could clear heat to relieve toxin, stop endogenous wind to arrest convulsion and clear away liver fire to improve eyesight [5,9,10].

#### Clinical indications

In traditional clinical practice, bear bile was used in fever fighting, detoxification, inflammation, swelling and pain reduction. It was also used in the cure of carbuncle of heat type, pyocutaneous diseases, hemorrhoid, overabundance of liver-fire, convulsion caused by the overabundance of heat, epilepsy, tic, and redness of eyes due to liver heat etc[5].

#### Application

For seasonal febrile disease with high fever in children and infantile convulsion, bear bile can be dissolved in milk or succus from Bambusae (Zhulishui in Chinese). For heat-toxin syndrome manifested by skin carbuncle, sore throat and hemorrhoids, bear bile can be used as oral administration or external use. In treating conjunctivitis and nebula, it is prepared to be a water solution as eyewash or used together with Borneolum (Bingpian in Chinese) [5]. Besides traditional use, the modern application of bear bile was extensive spread to many modern diseases based on traditional indications and pharmacological studies (also see Pharmacological study of bear bile and Scientific research).

#### Usage and dosage

0.25–2.5 g is taken as a dose in pill or powder. It is mainly single use and rarely used together with other herbs. Due to its fishy and bitter taste that may induce vomiting, it should be given as capsules. For external use, the fine powder can be applied to the local area or used with water [5].

#### Notes

Being extremely bitter and cold, it is easy to injure yang-qi of the spleen and stomach and so, it is contraindicated for deficiency and cold of the spleen and stomach [5].

### Modern research of bear bile

#### Chemical analysis of bear bile

Over the years, extensive research has been done in establishing methods to analyze and quantify the total and individual bile acids and their glycine and taurine conjugates in serum [11,12], pharmaceuticals [13,14], and bile [15-17], including bear gall drainage [18]. These methods mainly consisted of thin layer chromatography [13,18,19] and high-performance liquid chromatography [11,12,14-17,20,21].

Modern chemical research indicated that bear bile was mainly composed of bile acids, amino acids, bile pigments, fat and a few phospholipids and trace metals [22,23]. Bile acids were regarded as the principle components of bear bile in medical applications. It contained ursodeoxycholic acid (UDCA), chenodeoxycholic acid (CDCA), and cholic acid (CA). They usually occurred in form of conjugates solely with taurine in N-acyl linkage [22,24,25]. UDCA (3 alpha, 7 beta-dihydroxy-5 beta-cholan-24-oic acid) was found to be the primary bile acid produced in the liver of bears [26,27] and the relative high content of UDCA (1–39%) in bear (Ursidae) bile made it different from the ones originated from pandas, related carnivores (< 0.5%) [28] and other vertebrates [29].

It is important to note that variation of bile acid composition in bear bile was present. Espinoza EO *et al*. (1993) [24] reported that the bile originated from farmed bears (bears with chronic biliary fistulae, See also *Section 4.2 Extracted bear bile powder*) was characterized by a decrease in the percent composition of tauro-cholic acid (TCA) and a dramatic increase in that of tauro-ursodeoxycholic acid (TUDCA) and tauro-chenodeoxycholic acid (TCDCA). However, results of later investigation [30] indicated that the low content of TCA was common among wild and farmed Asian bears, while the samples from Polar and North American black bears showed a relative high content of TCA.

#### Pharmacological study of bear bile

Modern pharmacological research showed that bear bile has the following pharmacological actions: (1) antimicrobial and anti-inflammatory effect, (2) anti-hepatotoxic effect, (3) choleretic lithagogic effect, (4) anti-liver fibrosis, anti-tumor, anti-pyretic and sedative effect, (5) anti-convulsant and analgesic effect, (6) lowering lipid and hypotensive effect, (7) anti-tussive and anti-asthmatic effect, (8) improving eyesight, (9) anti-stress and relaxing effect [22,31].

As an anti-inflammatory drug in its clinical utilization, the suppressive effect of bear bile on inflammation was also investigated in extensive non-clinical studies. Using the model of rat granulation and feet swelling, it was reported that bear bile can significantly suppress inflammation in animal models. Moreover, it was also observed that bear bile can reduce the permeability of capillary vessel [32,33].

Recently, an investigation has been conducted to investigate the inhibitory effect of bear bile powder on rat liver fibrosis induced by dimethylnitrosamine (DMN). The results suggested that the bear bile powder could prevent the formation of liver fibrosis induced by DMN. The mechanism might relate to its inhibitory effect on the activation of Kupffer cell (KC) and hepatic stellate cell (HSC) [34]. Another study investigated the effect of tauroursodeoxyecholic acid (TUDCA), the characteristic compound of bear bile, on activity of glutamic pyruvic transaminase (GPT), and observed that TUDCA could significantly reduce GPT activities in mice serum and the inhibitory effect is independent to the concentration of substrate. No toxicity of TUDCA could be observed in this study [35]. These research demonstrated bear bile's clinical function as a hepatoprotective agent.

#### Clinical study of bear bile

Since bear bile has a wide range of pharmacological actions with little toxicological side effect, it is now being used clinically for curing hepatic and biliary disorders, cardiovascular and cerebrovascular disorders, pulmonary disorders, and ophthalmological disorders, while the pure compound derived from bear bile, UDCA, is widely used for hepatic and biliary disorders (also see 5.3 Scientific research). Bear bile as well as its Chinese Medicine formulae could also be applied to diabetes, nephritis, chincough, hemorrhoids, chronic hepatitis B induced jaundice, and falling sickness, etc. [22,31,36].

### Resource for bear bile

#### Natural bear bile

Traditionally, bear bile (Xiong Dan in Chinese) was the dried gall bladders of Tibetan brown bears (*Ursus arctos pruinosus*) or Asiatic black bears (from North-Eastern and Northern part of China) under the family of Ursidae. Later on, gall bladders from American black bears, Sun bears and Himalayan brown bear (*Ursus arctos isabelinus*) were also used in TCM [33].

The bear bile could be further divided into two types, namely, "Yun Dan" and "Dong Dan" in commercial market of TCM. The quality of the former was considered to be better. Gall bladders might be sold in whole pieces, in shavings, or in powder form which was put into capsules.

#### Extracted bear bile powder

Long time ago, people got bear bile via hunting activities but nowadays, people are used to getting bile from living bears. In the 1980's, The People's Republic of China has developed bear farms where over 7,000 brown bears and Asiatic black bears were kept in small cages. An un-sterile latex or stainless steel catheter was inserted through the external fistula directly into the gall bladders of each bear to drain the fluid daily either by gravity into a tray or by suction with an un-sterile syringe. This extraction method was called the "Free-dripping Fistula Technique". The fluid was then dried and manufactured as "Bear Bile Powder" (Bear bile extraction) [37]. The bears were suffering extreme pain due to daily bile extractions. Many of them often die from illnesses (such as cholecystitis, cholelithiasis, polyp formation, obstruction of the cystic duct, strictures and partial herniation of the gall bladder wall, liver cancer) and chronic infections caused by the presence of foreign bodies and their open wounds [38,39].

Products made from farmed bear bile included powder, capsules, ointments, tablets, tinctures, wines, suppositories, eye drops and bile tea. Bear bile powder was the most common type of product. It was usually produced and packaged by the bear farms themselves.

Comparative studies in chemical components of natural bear bile and bile extraction have been done in the past years and the results showed that both of the natural bear bile and the bile extraction contained similar chemical components and the quality of natural bear bile was much better [40-44].

#### Bear bile in the world

Trading of gall bladders from wild bears has been extensive over the past few decades. Tens of thousands of bears have been killed in the wilds to obtain their gall bladders and body parts, such as the paws (a delicacy in some Eastern countries), hide, claws, meat, fat and bones. Moreover, gall bladder has been the prize as it has the greatest commercial value. It was because prices of bear parts in China have dramatically risen. In 1970 one kilo of bear gall bladder cost around US $200, but by 1990 the price had risen to between US $3,000 and US $5,000 per kilo [45]. Recent market price with legal certification has risen to between US $30,000 and US $50,000 per kilo (our experience in legal market of Hong Kong). This has led to an increased threat to bears in the past few decades as price for bear gall bladder has increased making it a lucrative trade for hunters and middlemen alike.

Illegal products containing bear bile were on sale in Traditional Medicine shops in USA, Canada, Japan, Taiwan, Korea, Singapore, Australia and New Zealand according to the new findings from investigations led by the World Society for the Protection of Animals (WSPA) [46]. The report showed bear products offered by these shops were originated from China's bear farms.

With the great demand for bear bile products, fraudulent products were found in the markets. Two surveys reported that most of the bear bile products were indeed gall bladders or bile originated from domestic pigs, goats, and water buffalo or mixture with the true bear bile. Only minority of the specimens was found purely from bears [24,30].

### Strategy for stopping the trading of bear bile

From killing bears to get bear bile to adopting bears to extract bear bile, it failed to protect bears; on the contrary, cruelly put them in more pity position.

#### Roles of non-governmental organizations (NGOs) and mass media

Non-governmental organizations (NGOs) and the public could also play important roles on this issue, such as arousing public awareness, sharing of survey information that might assist governments, and raising the profile of this issue during communications with national authorities and international bodies. WSPA and its global network of member societies were working hard in requesting the Chinese Government to close down the bear farms.

In July 2000, an official signing ceremony among Animals Asia Foundation (AAF), the China Wildlife Conversation Association in Beijing and the Sichuan Forestry Department of China present, took place in public in Hong Kong where the "China Bear Rescue" agreement was announced to the worldwide press. The Agreement Summary was as follows [47]:

(1) The short-term goal was to close down the worst farms in Sichuan, China and to rescue 500 bears; this goal had been reached by end of March, 2008.

(2) The medium-term goal was to expand this program across China and continually reduce the number of bears on farms; and

(3) Finally, the long-term goal was to end bear farming and provide care and refuge for the remaining number of bears.

In this agreement, effort was given in supporting the research about the manufacture and the use of herbal and synthetic substitutes to bear bile and, in encouraging all consumers to refuse the use of any products containing bear bile. The Chinese officials would continue to maintain her policy of prohibiting the export of bile internationally. They would also launch public education programmes on the protection of wildlife and to preserve and protect the natural habitat of bears.

The AAF had agreed to pay a certain level of compensation to the farmers in an effort to ensure that none of these bears were slaughtered anymore for their parts. At the same time, it helped those affected bear farmers moved into alternative areas of employment. The Sichuan government passed the original copies of all confiscated licenses to AAF and a countrywide policy was set up in China to ensure that no new bear farm license would be issued.

However, bear farming still existed and WSPA believed there was no humane method of bile extraction. The farming bears for bile should be stopped as soon as possible due to the extreme animal cruelty involved and the negative effects on wild bear conservation.

To stop the illegal bear products trade and to protect wild bear populations, collaborations between CITES (Convention on International Trade in Endangered Species of Wild Flora and Fauna), national and local governments were needed to ensure that effective regulations and legislation were founded and executed.

In addition, direct communication with Chinese government was necessary to determine her long-term strategy for the bear farms, and to explain the reasons why bear farming should be ended.

If the demand for the bear bile products diminished, then illegal trade would stop automatically. Thus, public and consumer awareness programs should be carried out in key countries. Educational materials for the Traditional Medicine communities, including practitioners, traders and consumers need to create awareness of the wildlife conservation and animal welfare issues associated with the bear products.

#### Government policy

As the major production country of bear bile products, the Chinese government implemented some measures to protect the bear. In 2001, Ministry of Health announced that health products made of bear bile powder would not be approved any more in order to protect the sources of wild animals and guarantee the safety of health products. Since then, no such product was approved by Ministry of Health.

In 2005, regulations on the usage of bear bile in Chinese Medicine products were stipulated by the Chinese government. Food and Drug Administration of all provinces, and municipality directly under the Central government should strictly adhere to the regulations by limiting the usage of bear bile powder in Chinese Medicine products. Manufacturers of these products should submit their applications to their local Food and Drug Administration by listing out their categories, number of production and materials used, especially if there was bear bile powder.

As a matter of fact, it became a dilemma of whether bear bile should be used or not. The usage of bear bile was a problem of history, culture and economy and it might also become a political issue. On the one hand, there was pressure from wild animal protection groups on the Mainland and overseas. On the other hand, bear bile was an important raw material of TCM. No alternative materials could replace its integrative effects of pain-killing and anti-inflammatory for the time being. By far, there were 123 Chinese medicine products made of bear bile and 183 enterprises supported by bear bile powder [48]. Facing these two challenges, if bear farming industry had to be eliminated within China, an alternative for bear bile should be found out and widely promoted for producing Chinese Medicine products.

#### Scientific research

To look for an alternative of bear bile, a systematic research has to be done. The definition of "alternative" was a kind of material, which originated from animals or plants that were not classified as endangered species. It should also have medicinal effects similar to that of bear bile and could totally replace it in terms of clinical use.

In fact, some research has been done on alternative for bear bile from three aspects including: (1) animal drug substitutes, (2) pure chemical substitutes, and (3) plant drug substitutes. However, it would not be able to stop using bear bile immediately even an alternative was available. It was because it needs a long time to promote this alternative and gain acceptance from people by changing their culture, economic system and medical practice.

#### Comparative study of other animal biles

The bile acid compositions of several fowls and domestic animals, including toad, rabbit, chicken, pig and cattle, were preliminarily studied [29]. The results indicated that toad bile mainly composed of CA. In chicken bile, there were both CDCA and CA. Deoxycholic acid (DCA) was found in rabbit bile, while hyodeoxycholic acid (HDCA), lithocholic acid (LCA) and CDCA were present in pig bile. The composition of cattle bile was characterized by CA, DCA and minor CDCA.

The studies on bile of rabbits and pigs have been extensively carried out in comparison with those from other animals.

It was reported that both bear and pig bile solutions were shown to have anti-inflammatory, anti-convulsion and analgesic effects. They could also prolong the survival time of mice under hypoxic conditions. It was possible that pig gall bladders could be developed as an alternative for bear gall bladders in certain prescriptions of Chinese medicine [49].

Comparison between pharmacologic actions of rabbit bile and bear bile was also carried out. It was reported that many pharmacologic actions of the rabbit bile were similar to those of bear bile. It was also demonstrated that the effects of the rabbit bile were more obvious than those of bear bile in positive inotropic action, sedation, anti-tussive action, and anti-histaminic action, etc. [50].

These two kinds of gall bladders were generally considered to be able to substitute bear bile based on their chemical and pharmacological effects.

#### Pure chemical substitutes

Among the chemical components in bear bile, bile acids were the main effective components, which consisted of UDCA and CDCA. These two bile acids were a pair of isomer and the former made bear bile different from other animal biles. In other words, bears were the only mammals to produce significant amounts of UDCA (apart from giant pandas, which do not produce UDCA). It was also the UDCA which gave bear bile beneficial medicinal effects.

Although UDCA was also found in other animal species and could be extracted from pig bile and cow bile, the quantity was too little for large scale production. Japanese scientists succeeded in chemically synthesizing UDCA in 1955 [51]. Today, large quantities of UDCA were made synthetically and widely used in Western medicine to dissolve gallstones. It was estimated that 100,000 kg of this synthetic UDCA was being consumed each year in China, Japan and South Korea, and that the world consumption may double this figure.

Oral administration of either UDCA or CDCA could desaturate bile and contribute to the dissolution of cholesterol gallstones. However, the former was found to be better tolerated with fewer adverse side effects in comparison with the latter. In addition, recent evidence suggested that UDCA might be an effective drug in various chronic liver diseases, especially cholestatic disorders such as primary biliary cirrhosis [52].

A review paper was found to explain the rationale supporting the use of UDCA for the treatment of patients with cholesterol gallstones and chronic liver diseases and to describe the results obtained in clinical trials. The conclusion was that UDCA was a safe and effective drug for the treatment of patients with cholesterol gallstones [53].

#### The substitutes from Chinese herbs

In TCM, bear bile was classified under the category of clearing heat and detoxification drugs. In respect to the same medicinal functions, some Chinese herbs were also found to have the efficacies of clearing heat and detoxification, purifying liver and improving eyesight. It was suggested that one or more of these herbal medicines could be used as an alternative for bear bile [10,54,55]. A recent study on herbal alternatives to bear bile reported *Scutellaria baicalensis *Georgi, Huangqin in Chinese, and its active components, chrysin and wogonin, exhibited inhibitory effect on IL-6 promoter activity, indicating its potential as an anti-inflammation agents and an alternative to bear bile, traditional anti-inflammatory agent [56], but it is hard to say that Huangqin is a good candidate for substitute of bear bile because bear bile has multiple pharmacological action.

According to TCM herbal classification and the survey conducted for Chinese Medicine Practitioners by World Society for the Protection of Animals in Australia, Canada, USA, and UK, etc., Chinese herbs with the efficacies of clearing heat and detoxification included: *Lobeliae chinensis, Herba cum Radice*; *Oldenlandiae Diffusae, Herba*; *Patriniae seu thiaspi, Herba cum Radice*; *Pulsatillae Chinensis, Radix*; *Dictamni dasycarpi, Cortex Radicis*; *Andrographis paniculatae, Herba*); *Sedi sarmento, Herba*; *Isatidis, Folium*; *Sargentodoxae, Caulis*; *Lonicerae Japonicae, Flos*; *Forsythiae suspensae, Fructus*; *Lasiospherae seu calvatiae, Fructificatio*; *Portulacae oleraceae, Herba*; *Taraxaci mongolici*, *Herba cum Radice*; *Indigonis, Pulvis*; *Sophorae subprostratae, Radix*; *Belamcandae chinensis, Rhizoma*; *Houttuyniae cordatae, Herba cum Radice*; *Paridis, Rhizoma*, and *Violae yedoensitis, Herba cum Radice*.

Chinese medicines under the category of plant with the efficacy of clearing heat included: *Gentianae scabrae, Radix*; *Aloes, Herba*; *Paridis, Rhizoma*; and *Violae yedoensitis, Herba cum Radice*.

Chinese medicines under the category of plant with the efficacy of purifying liver and improving eyesight included: *Plantaginis, Semen*; *Lycii chinensis, Fructus*; *Eriocaulon buergerianum*, *Spica*; *Cassiae torae, Semen*; *Chrysanthemi morifolii, Flos*; *Viticis, Fructus*; *Buddleiae officinalis, Flos Immaturus*; *Equiseti hiemalis, Herba*; *Ligustri lucidi, Fructus*; *Citri reticulatae viride, Pericarpium*; *Celosiae argenteae, Semen*; *Mori albi, Folium*, and *Prunellae vulgaris, Spica*.

Other Chinese medicines under the category of plant with the efficacy of protecting liver and improving eyesight included: *Astragali complanati, Semen*; *Lycii chinensis, Fructus*; *Ligustri lucidi, Fructus*, and *Dendrobii, Herba*.

Although bear bile had the efficacy of clearing heat and detoxification, purifying liver, improving eyesight, and could be used to treat eclampsia, epilepsy, tic, hot liver, red eyes and swollen problem, it did not have the functions of protecting liver and could not be used to treat visual disturbance caused by weak liver and kidney according to TCM theory. As a result, scientists in pharmacology of Chinese medicine and Chinese Materia Medica were required to work together with TCM practitioners closely in order to find an alternative for bear bile. In other words, experts were required to carry out detailed research work and to propose scientific research methodology. Afterwards, stringent implementation should be ensured for proving the similar efficacies and mutual substitutability of the alternative.

#### Coptis as a herbal alternative to bear bile

Among the possible alternatives for bear bile, *Coptis *("Huanglian" in Chinese) was a possible choice since both of these materials were under the category of clearing heat. Apart from that, both bear bile and *Coptis *could detoxify, purify liver, improve eyesight, as well as treating eclampsia, epilepsy, tic caused by fire, carbuncle of heat type, pyocutaneous diseases. At present, prescriptions with *Coptis *(such as Huang Lian Jie Du Tang, Qing Ying Tang, and An Gong Niu Huang Wan) were widely used to treat symptoms such as coma, eclampsia, epilepsy, tic and we have also used it to treat liver diseases (Major active compound, UDCA derived from bear bile is mainly used for liver diseases) and cancer in TCM clinical practice[57]. Now, we are doing comparative research between bear bile and *Coptis *in chemical analysis, pharmacological study and clinical efficacy to clarify what we concern.

##### Natural resources of Copis

According to China Pharmacopeia (Edition 2005), three species of plants are regarded as the genuine resource of Coptis for medical utilization, Coptis chinensis Franch, Coptis deltoidea C.Y. Cheng et Hsiao and Coptis teeta wall. All these three species can be found in Mainland China. It was reported that Coptis teeta wall mainly grows in Yunnan Province of China, therefore it is named as Yunhuanglian in Chinese. Meanwhile, Coptis chinensis Franch (Chuanhuanglian or Weilian in Chinese) and Coptis deltoidea C.Y. Cheng et Hsiao (Yalian) grow in Sichuan Province. However, some other species are also used as resources of Coptis, such as Coptis omeiensis (Chen) C.Y. Cheng and Coptis chinensis Franch.var. brevisepala Wang et Hsiao [58].

##### Brief history of the traditional use of Coptis

Coptis (Huanglian) was firstly recorded in, <Shen Nong Ben Cao Jing>, a medical classic written in Han Dynasty, and was selected as a herbal drug for clearing heat and scavenging toxics. Moreover, in the ancient pharmacopeia <Ben Cao Gang Mu> in Ming Dynasty, Coptis and its formulations were used as therapeutic agents for eye diseases and diarrhea. A recent study summarized that Coptis in single dose form was most commonly used for the treatment of eye diseases in some ancient medical books (comprising 14.58% of the total records) [59]. Considering that optical system has close relationship with liver according to Chinese Medicine theories, these records indicates that Coptis may function to benefit liver organ, which is also regarded as parts of the pharmacological effect of bear bile in its traditional utilization. These links provide some evidences even inspirations for the use of Coptis as an alternative to bear bile. In terms of clinical applications, it was surprising to find the unexpected similarities between *Coptis *and bear bile, such as detoxify, purify liver, improve eyesight, as well as treating eclampsia, epilepsy, tic caused by fire, carbuncle of heat type, pyocutaneous diseases, sore throat and hemorrhoids.

##### Chemical composition and pharmacological actions of Coptis

The chemical composition of Coptis is complicated and dependent on species of samples. However, some common components amongst different species have been isolated and identified, including berberine, coptisine, palmatine, jatrorrhizine and magnoflorine[60]. In China Pharmacopeia (Edition 2005), berberine is used as a criterion for quality control of the Coptis. Thin layer chromatography and high performance chromatography analysis on berberine provide qualitative and quantitative evaluation to the herbs. Nonetheless, single criterion has its limitation in quality control system. Studies in our group have established, using HPLC-MS/MS, the chemical profile of Coptis and have made comparison on the chemical composition amongst species (data not shown). Differences can be easily observed from chromatograms so this provides a direct but comprehensive approach for the quality control of Coptis.

Extensive studies exhibited that Coptis has many pharmacological actions with strong clinical implications, including antibacterial, antiviral, antiinflammatory, antineoplastic, antihypertensive, antioxidative, antihyperglycemic and cholesterol-lowering effects [61-68]. Bioavailability of pure compound berberine derived from coptis is variety, traditionally it has been thought that berberine is poorly absorbed, but it has been reported that absorption in the mouse and human is good [69-71]. Previous studies in our group displayed that Coptis and its major component berberine showed promising potential as drug candidates for the treatment of liver injury [[72] and unpublished data]. It was also found from our *in vitro *study that Coptis and berberine can suppress cancer cell lines [[73] and unpublished data]. These positive data add new pharmacological activities for coptis and berberine and indicate the potential of coptis and berberine as an alternative to bear bile. Now comparative study on coptis and bear bile is our ongoing PhD program training project.

## Conclusion

To summarize, it was believed that the usage of bear bile is a problem of history, culture and economy and it may also become a political issue. Before stopping use of bear bile, it needs combined efforts from various routes of the society. As we reviewed in this paper, research on alternative for bear bile has been conducted in the past decade including comparative studies of various animal biles, the major effective components of bear bile, and substitutes of bear bile by artificial materials and herbal medicines. In this review, it showed that animal biles, such as those from pigs and rabbits, the synthetic compound of UDCA, and herbs, such as *Coptis*, were possible substitutes for bear bile. However, plant substitutes were only suggested in literature without solid evidence to support, while some animal substitutes were superficially studied. Thus, it is necessary for us to design systematic, serious and comparative research to get convincing data. It is important to note that animal substitutes will be found contradictory to the tenets of World Animal Protection sooner or later. The final choice will be using plant materials to substitute bear bile. Our previous study showed *Coptis *was a promising drug due to its similarity to bioactivity characteristics of bear bile, therefore comparative study for both *Coptis *and bear bile will be carried out. More persuasive research has to be done to explore their integrative functions and prove their similarities to bear bile in terms of medicinal use.

## Abbreviations

TCM: Traditional Chinese Medicine; UDCA: ursodeoxycholic acid; CDCA: chenodeoxycholic acid; TCA: tauro-cholic acid; TUDCA: tauro-ursodeoxycholic acid; TCDCA: tauro-chenodeoxycholic acid; CA: cholic acid; DCA: Deoxycholic acid; HDCA: hyodeoxycholic acid; LCA: lithocholic acid; WSPA: World Society for the Protection of Animals; AAF: Animals Asia Foundation; CITES: Convention on International Trade in Endangered Species of Wild Flora and Fauna; NGOs: non-governmental organizations.

## Competing interests

The authors declare that they have no competing interests.

## Authors' contributions

FY and SY retrieve data of bear bile, other animal biles and Chinese herbs from databases of Medline, Pubmed, other databases and data analyses and drafted the manuscript. WN retrieves data in Chinese from database of China Journals Full-text Database and translated them into English. NKM analyzed and summarized chemical data from above database. FY, TSW, NT and TY conceived of the study, supervision of the work and revision of the manuscript. All authors read and approved the final manuscript.
